# New Jersey State Lunatic Asylum

**Published:** 1847-04

**Authors:** 


17. *New Jersey State Lunatic Asylum.*—It is expected that this Institution will be completed the ensuing fall. It is beautifully situated a short distance from Trenton, and the building, which is of stone, is well arranged and well built. A medical superintendent has not as yet been appointed.

The completion of this Asylum and the Butler Hospital, *with all modern improvements*, should awaken the attention of the managers of other Institutions. They must not suppose they can be sustained solely by their past reputation;—progress and improvement in the care of the insane is as much demanded by the spirit of the age, as in other branches of business.—*Amer. Jour. of Insanity.*

